# Evaluation of cardiac function by global longitudinal strain before and after treatment with sofosbuvir-based regimens in HCV infected patients

**DOI:** 10.1186/s12879-018-3426-9

**Published:** 2018-10-16

**Authors:** Maria Mazzitelli, Carlo Torti, Jolanda Sabatino, Greta Luana D’Ascoli, Chiara Costa, Vincenzo Pisani, Elena Raffetti, Salvatore De Rosa, Alessio Strazzulla, Alfredo Focà, Maria Carla Liberto, Ciro Indolfi, the CARDIAC study group, Giorgio Settimo Barreca, Francesco Saverio Costanzo, Daniela Foti, Giorgio Fuiano, Giuseppe Greco, Francesca Serapide, Elio Gulletta, Nadia Marascio, Maria Concetta Postorino, Maria Adelina Simeoni, Alfredo Focà, Maria Carla Liberto, Aida Giancotti

**Affiliations:** 1 Unit of Infectious and Tropical Diseases, Department of Medical and Surgical Sciences, “Magna Graecia” University of Catanzaro, Viale Europa, 88100 Catanzaro, Italy; 2 Cardiovascular Institute, Department of Medical and Surgical Sciences, “Magna Graecia” University of Catanzaro, Viale Europa, 88100 Catanzaro, Italy; 3 Unit of Hygiene, Epidemiology and Public Health, Department of Medical and Surgical Specialities, Radiological Sciences and Public Health, Viale Europa, 25123 Brescia, Italy; 4 Institute of Microbiology, Department of Health Sciences, “Magna Graecia” University of Catanzaro, Viale Europa, 88100 Catanzaro, Italy

**Keywords:** Cardiac function, HCV eradication, DAA treatment, Longitudinal study

## Abstract

**Background:**

Possible cardiotoxicity of sofosbuvir in humans has not been demonstrated yet. Also, since HCV can exert deleterious effects on hearth function, it is of interest to know whether HCV eradication provides any benefits using global longitudinal strain (GLS), a measure of left ventricular function more reliable than ejection fraction (EF).

**Methods:**

Patients eligible for treatment with the combination therapy for HCV were invited to perform a transthoracic cardiac ultrasound at four different time points: before starting treatment, after one month, at the end of treatment and, after six month. Left ventricular function was measured with both EF and GLS.

**Results:**

From March 2015 to December 2016, 82 patients were enrolled. Fifty-six percent patients were males. Mean age was 66.12 (SD: 9.25) years. About 20% patients did not present any cardiovascular risk factors or comorbidities. A worsening trend of GLS was observed. Variations were not found to be statistically significant when EF was studied along the follow-up. However, when GLS was studied, its variations were found to be statistically significant indicating a worsening effect, albeit with different trends in patients who underwent treatment for three months compared to six months. Worsening of GLS was found to be statistically significant even after adjusting for body mass index and liver fibrosis, independently from treatment duration.

**Conclusions:**

Our results showed unexpected worsening of left ventricular function when measured through GLS after HCV treatment response induced by DAAs including sofosbuvir. Although this result is not proven to be clinically significant, the safety profile of sofosbuvir-based regimens needs to be studied further.

**Electronic supplementary material:**

The online version of this article (10.1186/s12879-018-3426-9) contains supplementary material, which is available to authorized users.

## Background

Extra-hepatic manifestations (such as neoplastic, autoimmune and vascular diseases) occur in about 70% of patients infected by hepatitis C virus (HCV) [1–3]. Among these manifestations, cardiovascular diseases (CVD) are more prevalent in HCV infected patients, but mechanisms are currently unknown. HCV related inflammation, oxidative stress [4, 5] and direct damage due to HCV infecting cardiac cells [6–8] might have a impact.

Animal studies reported death for cardiac causes after administration of a sofosbuvir metabolite at blood concentrations much higher than the therapeutic index used in humans [9]. Currently there is a lack data on the effects of sofosbuvir on heart function. Such data are important to confirm safety of sofosbuvir because we are currently treating aging populations with a significant prevalence of heart diseases. On the other way round, it is possible that clearance of HCV with interferon-free regimens would act favourably, as it was previously demonstrated that HCV eradication with interferon based regimens is able to reduce mortality for cardiovascular events [10, 11].

Left ventricular function (LVF) is routinely evaluated through the ejection fraction (EF), calculated by means of the modified Simpson method in current clinical practice, with the use of trans-thoracic echocardiography [12]. Trans-thoracic echocardiography based speckle tracking assessment is more reliable and precise for the assessment of myocardial function than trans-oesophageal ultrasound [13].

More recently, the global longitudinal strain (GLS) was developed as a more reliable index to measure left ventricular function [14]. Indeed, GLS was shown to be a valuable clinical parameter and a independent predictor of all cause mortality in patients with CVD [15]. Moreover, variations of GLS have been found in diverse conditions such as doxorubicin-induced cardiomyopathy, HIV infection in children and young adults, or viral myocarditis [16–19]. In these conditions, even minor variations of GLS were clinically meaningful, even when EF seemed to be preserved [18, 19]. For instance, data showed that GLS provides incremental diagnostic and prognostic information, that are correlated with histological findings in patients with viral myocarditis for whom conventional 2D echocardiography is unspecific, particularly in those with a preserved EF [18, 19]. This correlation was independent from conventional 2D echocardio-graphic parameters showing that strain rate and strain imaging are more sensitive in the detection of early changes or mild myocardial damage. Moreover, patients with impaired strain rate and strain at the acute phase of the disease showed worse short-time echocardiographic outcomes. For these patients, clinical history, physical examination, ECG, and serology were shown to be unreliable compared with GLS.

## Methods

### Aim

In the present study, we aimed at measuring possible changes of cardiovascular function in patients with chronic HCV infection before and after sofosbuvir-based regimens, using both left ventricular EF and GLS. The latter was chosen as advanced biomarker to measure the effect.

### Population and data collection

We conducted a longitudinal study from March 2015 to January 2017, enrolling all HCV infected patients treated with sofosbuvir-based regimens at the outpatient clinic of “*Mater Domini*” teaching hospital in Catanzaro (Italy), according to the criteria set by the Italian Medicinal Agency (AIFA) (see Additional file 1: Table S1). For patients without clinical cirrhosis or extra-hepatic manifestations, transient elastography (FibroScan™) was performed in order to estimate liver fibrosis so as to ascertain indications for treatment.

Exclusion criteria were: age less than 18 years old, pregnancy, and severe chronic disease (estimated glomerular filtration rate, eGFR< 30 mL/min).

Patients were assessed at four time points: baseline (i.e., before treatment initiation), after one month, at the end of the treatment course (either month 3 or month 6), and after 6 months from the end of treatment (off treatment follow-up).

Cardiac ultrasound was performed at baseline and at each follow-up using trans-thoracic Vivid E9 ultrasound. Speckle tracking echocardiography analysis was performed from apical views. Standard grayscale 2D images were obtained at a frame rate of 70–90 frames/s during three cardiac cycles and software package (EchoPAC™, GE healthcare) was used for offline analysis. Two expert cardiologists (L.G.D.A. and J.S.) performed cardiac ultrasound blinded of previous examinations, type and length of prescribed treatments.

At baseline, risk factors for CVD (i.e., hypertension, diabetes mellitus, cigarette smoking, previous stroke or myocardial infarction were recorded), and heart diseases were carefully investigated. Patients were considered to be underweighted (BMI ≤ 18.4 Kg/m^2^), normal (BMI = 18.5–24.9 Kg/m^2^), over-weighted (BMI = 25–29.9 Kg/m^2^) or obese (BMI ≥ 30 Kg/m^2^) [20].

Complete blood count, AST, ALT, total and fractioned bilirubin, and HCV RNA were recorded at enrolment and each follow-up points. Indirect indices of fibrosis, such as Fibrosis 4 index (FIB-4) and AST to platelets ratio (APRI) score were calculated at baseline and at month 6 after the end of treatment [21–23]. At these time points, alpha-fetoprotein, cholesterol, creatinine, glucose, INR, triglycerides were also evaluated. Data were stored in an ad-hoc electronic database.

Drug interactions with other co-medications were carefully evaluated using the application HEP ​​Drug Interaction [24]. Drugs with a significant risk of interaction with antivirals were substituted. For example, after cardiological consultation, amlodipine was reduced from 10 mg to 5 mg per day in patients who received daclatasvir or ledipasvir, if possible, or otherwise substituted.

This study was coordinated by the Infectious and Tropical Diseases Unit in collaboration with the Cardiology Unit of “*Mater Domini*” teaching hospital in Catanzaro (Italy) and was conducted in accordance with the guidelines of the Declaration of Helsinki and the principles of Good Clinical Practice [25–27]. The local Ethical Committee (Calabria Region) approved the study protocol and written informed consent was obtained from all subjects enrolled.

### Statistical analysis

To adjust the analysis for treatment duration, the enrolled patients were ranked into two groups: group A, i.e. patients with indication for a 3 month treatment, and group B, i.e. patients with indication for a 6 month treatment with DAAs. Study parameters were expressed as means (standard deviation, SD) or proportions as appropriate. FIB-4, APRI score, alpha-fetoprotein, creatinine, cholesterol, glucose, haemoglobin, and triglycerides values at baseline were compared with those at last follow-up using Student’s t-test for paired data. We evaluated the temporal trends of AST, ALT, platelet count, total bilirubin, EF and GLS using univariate mixed models for repeated measures. We also assessed the temporal trend of GLS using a multivariate mixed model adjusting for BMI, fibrosis and duration of treatment (3 or 6 months). Moreover, although in the analysis hypertension was not a confounder by definition, since it could have been associated with the outcome (GLS) but not with the exposure, we tested whether hypertension was a effect modifier.

Lastly we explored whether ribavirin could have a role on the change of GLS over time using a mixed model with an interaction term between ribavirin and time.

All statistical tests were two-sided, assuming a level of significance of 0.05 and were performed using Stata software version 12.0 (StataCorp, College Station, TX, USA).

## Results

### Patient flow and characteristics

**Fig. 1:**
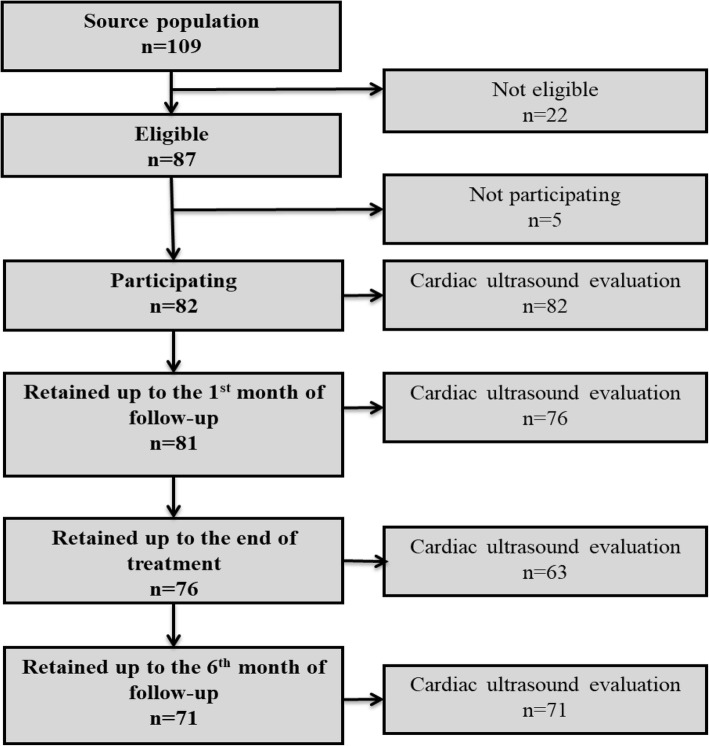
Flow chart of the patients along the study time points. Eighty-two patients decided to participate and underwent cardiac ultrasound at baseline. Fifty-seven patients were prescribed a 3-month treatment (group A) while 25 patients underwent treatment for 6 months (group B). Among patients who showed up at clinical checks, 76/81 presented to perform cardiac ultrasound at first month, 63/76 at the end of treatment and 71/71 at the last follow-up point

Among 109 patients who started a DAA treatment during the study period, 87 subjects were eligible and 82 were enrolled (56% males, mean age of 66.1 years). Amongst these patients, 71/82 (86.6%) continued follow-up until the end of the study (Fig. 1).

Seventy-two (87.9%) patients met AIFA criterion 1 or 4. Nine patients had extra-hepatic manifestations (AIFA criterion 3), and one patient had a HCV RNA relapse after liver transplantation (AIFA criterion 2). Fifty-seven (69.5%) patients were prescribed a treatment lasting for three months, while 25 (30.5%) were prescribed a treatment for six months. The baseline characteristics of patients are described in Table 1. With regards to CV risk factors, 59.3% patients were overweighed, 4.7% had a previous major cardiovascular event (stroke or myocardial infarction), 17.4% were smokers, 62.8% had hypertension and 26.7% had diabetes mellitus. Overall, only 20% of subjects did not present any CV risk factors or comorbidities. About 70% patients had previous experience to interferon-based regimens.

**Table 1:** Characteristics of the enrolled patients overall and by length of treatment (group A: treatment lasting for 3 months and group B: treatment lasting for 6 months)

Variable	Total n (%)	Group A n (%)	Group B n (%)	*p*-value
Gender				
Male	46 (56.1)	35 (61.4)	11 (44)	
Female	36 (43.9)	22 (38.6)	14 (56)	0.144
Age (years)				
≤ 60	22 (26.8)	17 (29.8)	5 (20)	
61–68	22 (26.8)	16 (28.1)	6 (24)	
69–74	24 (29.3)	15 (26.3)	8 (32)	
≥ 75	14 (17.1)	9 (15.8)	6 (24)	0.666
Type of liver disease				
No cirrhosis or HCC	45 (54.9)	34 (59.6)	9 (36)	
Cirrhosis	35 (42.7)	23 (40.4)	14 (56)	
HCC with cirrhosis	2 (2.4)	0 (0)	2 (8)	0.025
Transient elastography				
None	7 (8.5)			
F0	0 (0)	0 (0)	0 (0)	
F1	2 (2.4)	2 (3.9)	0 (0)	
F2	3 (3.8)	3 (5.9)	0 (0)	
F3	33 (40.2)	29 (56.9)	3 (13)	
F4	37 (45.1)	17 (33.3)	20 (87)	< 0.01
HCV Genotype				
1a	2 (2.4)	1 (1.7)	1 (4)	
1b	60 (73.3)	42 (73.7)	17 (68)	
2	6 (7.3)	3 (5.3)	3 (12)	
2a/2c	5 (6.1)	6 (10.5)	0 (0)	
3	4 (4.8)	1 (1.7)	3 (12)	
4	5 (6.1)	4 (7.1)	1 (4)	0.148
Co-infections				
None	81 (98.8)	56 (98.3)	25 (100)	
HIV +	1 (1.2)	1 (1.7)	0 (0)	
HBsAg +/HIV -	0 (0)	0 (0)	(0)	0.505
BMI				
Normal	23 (28)	18 (31.6)	5 (20)	
Overweight/Obese	59 (71.0)	39 (68.4)	20 (80)	0.283
Risk factors for CV diseases^a^				
None	19 (23.2)	15 (26.3)	4 (16)	0.308
Hypertension	51 (62.2)	34 (59.6)	17 (68)	0.473
Diabetes mellitus	21 (25.6)	12 (21.1)	8 (32)	0.288
Smoking habits	14 (17.1)	11 (19.3)	3 (12)	0.419
Previous CV events	4 (4.8)	3 (5.3)	1 (4)	0.807
Comorbidities^a^				
None	19 (23.2)	15 (26.3)	4 (16)	0.308
eGFR < 90 ml/min	43 (52.4)	32 (39)	11 (13.4)	0.350
Osteoporosis	15 (18.3)	10 (17.6)	5 (20)	0.791
Depression	14 (17.1)	11 (19.3)	3 (12)	0.419

^a^Each patient may have more than one risk factors and comorbidities

*CV* Cardiovascular, *eGFR* Estimated glomerular filtrate rate

### Treatment course

Prescribed treatments and related outcomes are described in Table 2. Most patients (97.56%) reached the end of treatment; only one patient stopped prematurely for a psychotic syndrome and another for virological failure. Seventy-nine (96.4%) patients gained sustained virological response (SVR) at weeks 12 after the end of treatment. Two patients (2.4%) had virological failure. All patients tolerated treatments very well, without any severe adverse events recorded. Among 49 patients who received ribavirin, folic acid and/or erythropoietin were added for anaemia in 11 (22.4%) and in 1/49 (2.04%) ribavirin was stopped for the same reason. Table 3 shows temporal trends of selected parameters. Liver parameters improved, whereas cholesterol rose in both groups (treatment length of 3 or 6 months).

**Table 2:** Prescribed treatment, supportive drugs and related outcome (*n* = 82)

Cate	n (%)
Prescribed DAAs	
SOF + RBV	10 (12.2)
SOF + SIM ± RBV	29 (35.4)
SOF + LDV ± RBV	31 (37.8)
SOF + DCV ± RBV	12 (14.6)
Ribavirin	
Yes	49 (59.7)
No	33 (40.3)
Ribavirin modification	
None	26 (53.1)
Reduction	22 (44.9)
Suspension	1 (2)
Adding support drug for anaemia in patients with ribavirin	
None	38 (77.6)
Folic acid	7 (14.2)
Erythropoietin	2 (4.1)
Folic acid + erythropoietin	2 (4.1)
Reason for stopping DAAs	
End of treatment	80 (97.6)
Patient decision	1 (1.2)
Virological failure	1 (1.2)

*DAAs* Direct antiviral agents, *SOF* Sofosbuvir, *RBV* Ribavirin, *SIM* Simeprevir, *LDV* Ledipasvir, *DCV* Daclatasvir

**Table 3:** Parameters at baseline and during follow-up

Parameters	Group A baseline mean (SD)	Month 1 mean (SD)	End of treatment mean (SD)	6^th^ month of follow-up mean (SD)	*p*-value*	Group B baseline mean (SD)	Month 1 mean (SD)	End of treatment mean (SD)	6^th^ month of follow-up mean (SD)	*p*-value*
FIB-4	3.8 (3.6)	–	–	2.7 (1.8)	0.003	5.5 (5.3)	–	–	3.7 (4.7)	< 0.001
APRI SCORE	1.2 (1.1)	–	–	0.6 (0.9)	< 0.001	1.7 (1.8)	–	–	0.9 (2.1)	0.002
INR	1.1 (0.2)	–	–	1.1 (0.1)	0.111	1.1 (0.2)	–	–	1.1 (0.1)	0.435
α-fetoprotein (ng/mL)	12.8 (20.9)	–	–	7.8 (19.3)	< 0.001	19.5 (28.9)	–	–	5.9 (3.1)	0.014
Creatinine (mg/dL)	0.8 (0.2)	–	–	0.9 (0.2)	0.031	0.7 (0.1)	–	–	0.8 (0.1)	0.032
Glucose (mg/dL)	112 (29)	–	–	110.5 (30.8)	0.266	128.6 (41.7)	–	–	119.3 (31.7)	0.337
Haemoglobin (g/dL)	14.2 (2.2)	–	–	14.1 (2.1)	0.551	13.6 (1.9)	–	–	13.9 (1.8)	0.625
Cholesterol (mg/dL)	155.2 (30.8)	–	–	163.5 (29.8)	0.070	149.9 (38.1)	–	–	179.3 (46.2)	0.003
Triglycerides (mg/dL)	111.25 (46.12)	–	–	110.96 (46.49)	0.155	112.12 (39.64)	–	–	100.8 (46.73)	0.295
AST (UI/L)	59.2 (40.9)	24.8 (13.3)	22.9 (9.1)	25.6 (13.4)	< 0.001	68.7 (40.6)	29.8 (19.7)	23.8 (8.3)	24.3 (8.3)	< 0.001
ALT (UI/L)	65. (41.3)	21.7 (11.4)	18.3 (6.8)	20.5 (9.4)	< 0.001	69.7 (52.3)	24 (15.1)	20.5 (8.5)	20.3 (10)	< 0.001
Platelet (×10 [3]/mL)	166.8 (65.8)	183.4 (78.5)	171.1 (77.5)	167.1 (61.5)	0.399	139.3 (62.1)	161.2 (72.3)	153.9 (65.4)	150.2 (58.9)	0.779
Total bilirubin (mg/dL)	0.9 (0.7)	1.1 (0.7)	0.9 (0.8)	0.7 (0.5)	< 0.001	1 (0.8)	1 (0.7)	0.7 (0.6)	0.8 (0.5)	< 0.001
Ejection Fraction (%)	56.5 (3.1)	56.9 (3.5)	56.6 (2.5)	56.7 (2.8)	0.499	56.9 (2.9)	57 (3)	57.2 (3.2)	57.4 (3.6)	0.535
GLS (%)	−20.8 (2.8)	−21.4 (2.4)	−20.9 (2.6)	−20.3 (2.6)	0.031	−21.1 (2.4)	−20.7 (2.7)	−20.3 (2.8)	−20.1 (2.5)	0.097

We compared baseline and 6th month of follow-up values of the two groups of treatment (group A = 3 months of treatment, group B = 6 months of treatment) with t-test for FIB-4, APRI SCORE, α-fetoprotein, creatinine, glucose, haemoglobin, cholesterol, and triglycerides. We use mixed-linear models to evaluate the linear trend of the other parameters

--: the parameter is not available at this follow-up point

### Evaluation of cardiac function

At baseline, mean EF and GLS were 56.7% and − 20.9%, respectively. Hence, 20/82 (24.1%) patients had abnormal EF (< 55%), while 3/82 (3.6%) had abnormal GLS (> − 16.5%) according to litereature standards [28, 29]. Compared to those with lower values, subjects with GLS ≥ median value of the study population (− 20.3%) had higher BMI (mean 27.9 vs. 26.0), higher haemoglobin (14.6 vs. 13.5 g/dL), higher triglycerides (123.5 vs. 98.3 mg/dL) and a greater proportion of current smokers was found (71.4% vs. 44.1%) (see, Additional file 1: Table S2). Moderate mitral and tricuspid insufficiencies were diagnosed in 2 patients.

**Fig. 2:**
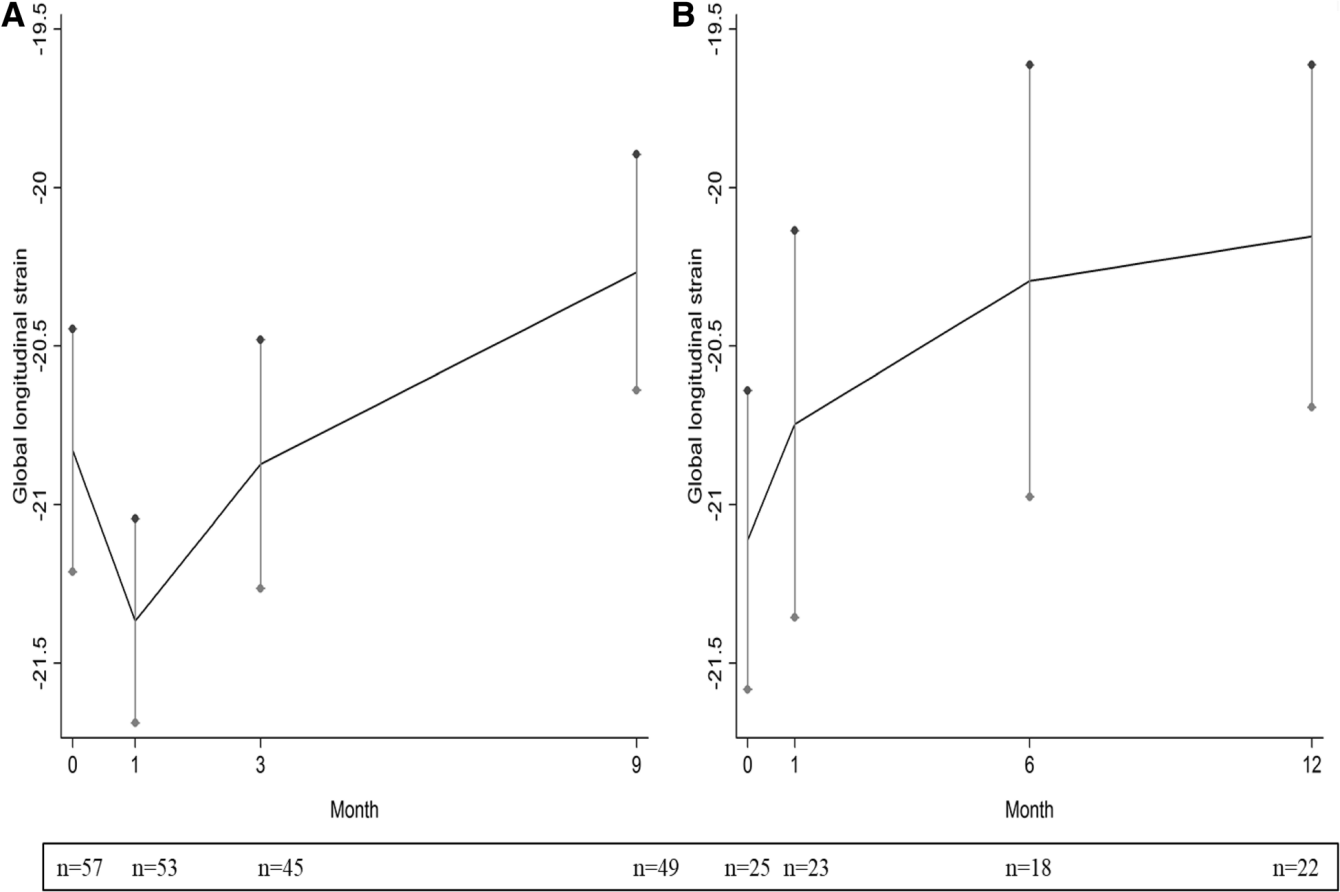
Distribution of global longitudinal strain (GLS) at baseline, 1st month, at end of treatment and at 6th month of follow-up after treatment in the two groups of patients by treatment length, either 3 months (group A) or 6 months (group B). At each time point, the number of patient who performed cardiac ultrasound is showed below the X-axis

As illustrated in Table 3, while there were not statistically significant variations of EF along the follow-up in both groups, a statistically significant worsening of GLS was found in the group of patients treated for three months (group A), while in patients treated for six months (group B) only a tendency towards a statistically significant worsening was found. Interestingly, GLS displayed a biphasic trend in the 3-month group, decreasing from − 20.8% at baseline to − 21.4% at month 1, before rising up to − 20.3% at the end of the follow-up (*p* = 0.031) (Fig. 2A). By contrast, GLS increased steadily from − 21.1% to − 20.1% in the six-month group (*p* = 0.097) (Fig. 2B). The rise of GLS over time was confirmed in a multivariate mixed model adjusted for BMI, liver fibrosis and treatment length with a mean GLS increase of 0.07 (0.01–0.13) per month (*p* = 0.013) (see, Additional file 1: Table S3).

Lastly, we explored whether hypertension was a effect modifier but we did not found any significant evidences (coefficient − 0.43, 95% CI: -1.42 to 0.55; *p* = 0.388). We also tested whether ribavirin could have a role on the change of GLS over time and in a mixed model with an interaction term between ribavirin and time but ribavirin exposure did not exert a statistically significant effect on GLS (absent ribavirin coefficient − 0.607, 95% CI: -1.608 to 0.395; *p* < 0.235) or a statistically significant role as effect modifier was not demonstrated (coefficient − 0.018, 95% CI: -1.69 to 0.39; *p* = 0.749).

## Discussion

The main finding of our study is that cardiac function measured through GLS seemed to worsen in the overall population, while EF did not change significantly. This may indicate that sofosbuvir based treatment could exert a negative impact on cardiac function. Possible toxicity of sofosbuvir may be supported from data showing that development of another NS5B polymerase inhibitor (BMS5986094) was stopped after a safety signal of cardiotoxicity [30]. In this work a young male died for rapidly progressive heart failure and 41.2% (14/34) patients had some evidence of cardiac dysfunction (6/14 with EF < 30% and 8/14 from 30 to 50%). So, as far as cardiotoxicity is concerned, a class effect of NS5B polymerase inhibitors should be studied further. Interestingly, after stopping DAAs, GLS continued to worsen, possibly indicating a prolonged effect. In addition, since EF remained stable, we may hypothesize that, similar to other conditions [14, 15], GLS is a more sensitive method to measure cardiac function.

However, the clinical significance and the long-term effects of the GLS variations in our patients are unknown. Correlations with other biomarkers of heart dysfunction (such as troponin, NT-pro-BNP, and micro-RNAs) [31–34] and long term studies with “hard” clinical end-points would be helpful. Also, it is difficult to explain why such an effect was demonstrated. In fact, besides a direct effect of sofosbuvir, other explanations may be found, including a random effect due to the small number of patients, an effect of concomitant drugs, or an indirect effect of HCV eradication mediated by inflammatory changes [4, 35]. So, the major difficulty that comes with the dataset studied herein is to dissect whether the effect can be ascribed entirely to sofosbuvir or to other factors. For this reason, more powerful studies should adjust for possible confounders (including concomitant drugs and co-morbidities such as hypertension or cholesterol level and its variations), and using immune parameters to provide more specific and detailed information from the pathogenic point of view. Also, we need studies with a different design to assess whether sofosbuvir or HCV eradication (and possible immune effects related to this eradication) are implicated. For instance, one could compare cardiac function in patients with sustained virological response with respect to those without response, or cardiac function may be evaluated in healthy volunteers. Moreover, a group of control patients with other aetiologies presenting the same risk factors, but not treated with sofosbuvir-based treatments would be helpful. Unfortunately, however, this is difficult (or unethical) to be accepted for the legitimate desires of patients to be treated as soon as possible.

Since we did not find any significant correlations between GLS and ribavirin or anaemia (data not shown), we may hypothesize that these factors were not implicated. However, we have to take into account that the small sample size reduced the power to detect a smaller effect of ribavirin, significantly. Indeed, the evidence of a prolonged worsening of GLS after stopping treatment is more consistent with an effect of ribavirin (whose multiple dose half-life is around 12 days, persisting in non-plasma compartments for as long as 6 months) than with an effect of sofosbuvir (whose half life is only 0.4 h). For the same reason, the trend in GLS is more consistent with an immune-mediated phenomenon occurring after viral eradication, so consideration of immune markers could provide better insights on the phenomenon. With regard to hypertension, we did not find any significant evidences of a possible role at interaction model independently from time, but a complete assessment in a multivariable model would require grater numbers and a time-dependent consideration of hypertension as a variable in future studies.

In patients treated for three months, we noted an initial improvement of GLS, followed by a progressive worsening. The first phase of improvement could be due to a beneficial reduction of HCV RNA [10] while apparent sofosbuvir toxicity or other negative phenomena may have become more evident afterwards. This biphasic trend was not evident in patients treated for 6 months. The fact that patients who received 6 months of treatment were older, more likely to present advanced liver fibrosis or cirrhosis and comorbidities (including cardiac ones) could explain the discordant trends of GLS in the two groups. In fact, healthier individuals could benefit more from HCV RNA clearance in the short-term, while more compromised patients may suffer from a more prompt cardiotoxicity of sofosbuvir. Thus, it is worth considering that extreme elderly patients are receiving DAA treatments, with a high SVR rate, but at the same time they may experience more frequent cardiovascular complications, therefore a close and accurate monitoring of heart function could be required [36–38].

The associations between worse GLS and smoking or high BMI at baseline was not unexpected, suggesting the importance to quit negative behaviours, such as smoking and unhealthy diet in patients chronically infected by HCV. This is even more relevant if one considers that cardiac function may worsen after treatment, concomitantly with an increase of cholesterol occurring after HCV eradication as demonstrated in our study and confirmed by others [39]. Appropriate time dependent analysis should be conducted to assess whether variations in cholesterol levels may lead to GLS changes during SOF-based regimens.

## Conclusions

In conclusions, if confirmed by datasets from independent cohorts to replicate the data, our results are important because demonstrated for the first time the possible cardiotoxicity of DAA treatments. The same study protocol for patients who are eligible for DAAs treatment with sofosbuvir-free regimens should be applied, in order to evaluate whether worsening of GLS is a specific drug-related or a class effect. While these results should be confirmed in more powerful studies and pathogenic hypotheses should be tested in translational studies, in the meantime a cautious approach should include assessment of cardiac function during DAA treatment, particularly for the most fragile patients, who may benefit from interventions to reduce the risk of cardiovascular diseases both before and after treatment.

## Additional file

Additional file 1: **Table S1**. AIFA (Italian Regulatory Agency for Drug Administration) criteria for prescription of a DAA treatment. **Table S2**. Association between global longitudinal strain (dichotomized on median) and demographical and clinical features at baseline. **Table S3.** Multivariate mixed model, effect of time, BMI, presence of significant liver fibrosis and duration of treatment on global longitudinal strain. (DOCX 24 kb)

## Abbreviations

AIFA: Italian medicinal agency
APRI: AST to platelets ratio
BMI: Body mass index
CVD: Cardiovascular diseases
DAAs: Direct antiviral agents
DCV: Daclatasvir
EF: Ejection fraction
eGFR: Estimated glomerular filtrate rate
FIB 4: Fibrosis 4 index
GLS: Global longitudinal strain
HCV: Hepatitis C virus
LDV: Ledipasvir
LVF: Left ventricular function
RBV: Ribavirin
SD: Standard deviation
SIM: Simeprevir
SOF: Sofosbuvir
SVR: Sustained virological response
