# Supplementary material for: Evaluation of cardiac function by global longitudinal strain before and after treatment with sofosbuvir-based regimens in HCV infected patients
Source: BMC Infect Dis. 2018 Oct 16;18:518. doi: 10.1186/s12879-018-3426-9 (PMC6192073; doi:10.1186/s12879-018-3426-9)
Supplement: Supplementary file 1 — Table S1. AIFA (Italian Regulatory Agency for Drug Administration) criteria for prescription of a DAA treatment. Table S2. Association between global longitudinal strain (dichotomized on median) and demographical and clinical features at baseline. Table S3. Multivariate mixed model, effect of time, BMI, presence of significant liver fibrosis and duration of treatment on global longitudinal strain. (DOCX 24 kb) [file 12879_2018_3426_MOESM1_ESM.docx]

**Table S1**. AIFA (Italian Regulatory Agency for Drug Administration) criteria for prescription of a DAA treatment

| AIFA criteria |
| --- |
| 1. Patients with CHILD A or B cirrhosis and/or HCC with complete response to surgical resettive or loco-regional therapy, not eligible for liver transplantation, in which liver disease significantly affects the prognosis 2. Patients with positive HCV-RNA recurrent hepatitis of the liver graft, clinically stable and with optimal level of immunosuppression 3. Patients with chronic hepatitis with extra-hepatic HCV-related manifestations (cryoglobulinemic syndrome with organ damage, B-cell lymph-proliferative syndrome) 4. Patients with chronic hepatitis with fibrosis METAVIR F3 (or corresponding ISHAK) 5. Patients in the listing for hepatic transplantation with cirrhosis MELD <25 and/or HCC inside of Milan criteria with the possibility of waiting for at least 2 months list 6. Patients with chronic hepatitis after solid organ transplantation (not liver) or with METAVIR marrow fibrosis ≥ 2 (or corresponding ISHAK) |

**Table S2**. Association between global longitudinal strain (dichotomized on median) and demographical and clinical features at baseline

| Variables | GLS ≤ -20.3%  n (%) | GLS > - 20.3%  n (%) | p-value |
| --- | --- | --- | --- |
| Qualitative  Gender |  |  |  |
| Male | 21 (45.6) | 25 (54.3) | 0.254 |
| Female | 21 (58.3) | 15 (41.5) |  |
| Age |  |  | 0.896 |
| ≤ 60 years | 10 (45.45) | 12 (54.5) |  |
| 61-68 | 12 (54.55) | 10 (45.4) |  |
| 69-74 | 12 (50) | 12 (50.0) |  |
| ≥ 75 | 8 (57.1) | 6 (42.9) |  |
| Liver disease |  |  | 0.367 |
| No cirrhosis/no HCC | 22 (48.9) | 23 (51.1) |  |
| Cirrhosis | 18 (51.4) | 17 (48.6) |  |
| HCC | 2 (100.0) | 0 (0.0) |  |
| Hypertension Yes | 28 (54.9) | 23 (45.1) | 0.392 |
| No | 14 (45.2) | 17 (54.8) |  |
| Diabetes Yes | 10 (47.6) | 11 (52.4) | 0.702 |
| No | 32 (54.5) | 29 (47.5) |  |
| Previous CV events Yes | 2 (50.0) | 2 (50.0) | 0.960 |
| No | 40 (51.3) | 38 (48.7) |  |
| Cigarette’s smoking Yes | 4 (28.6) | 10 (71.4) | 0.063 |
| No | 38 (55.9) | 30 (44.1) |  |
| Depression Yes | 6 (42.9) | 8 (57.1) | 0.492 |
| No | 36 (52.9) | 32 (47.1) |  |
| Osteoporosis Yes | 7 (46.7) | 8 (53.3) | 0.696 |
| No | 35 (52.2) | 32 (47.8) |  |
| Quantitative  FIB-4 index, mean (SD) | 4.7 (0.7) | 3.9 (0.6) | 0.403 |
| APRI score, mean (SD) | 1.5 (0.2) | 1.3 (0.2) | 0.365 |
| BMI, mean (SD) | 26.0 (0.5) | 27.9 (0.6) | 0.019 |
| Ejection fraction, mean (SD)  Serological parameters | 56.2 (0.5) | 57.1 (0.4) | 0.164 |
| α- fetoprotein ng/dL, mean (SD) | 19.2 (4.9) | 11.6 (1.8) | 0.161 |
| Cholesterol mg/dL, mean (SD) | 150.7 (5.5) | 155.1 (4.7) | 0.551 |
| Creatinine mg/dL, mean (SD) | 0.79 (0.02) | 0.82 (0.03) | 0.431 |
| Hemoglobin g/dL, mean (SD) | 13.5 (0.3) | 14.6 (0.3) | 0.019 |
| Triglycerides mg/dL, mean (SD) | 98.3 (5.5) | 123.5 (8.7) | 0.015 |

**Abbreviations**: GLS, global longitudinal strain; HCC, hepatocellular carcinoma; FIB-4, Fibrosis 4 index; SD, standard deviation; BMI, body mass index; CV, cardiovascular.

**Table S3.** Multivariate mixed model, effect of time, BMI, presence of significant liver fibrosis and duration of treatment on global longitudinal strain

|  | **Coefficient** | **95% CI** | **P value** |
| --- | --- | --- | --- |
| Time (months) | 0.07 | 0.01, 0.13 | 0.013 |
| BMI | 0.22 | 0.11, 0.34 | <0.001 |
| Liver fibrosis (Yes vs No) | 0.1 | -0.56, 0.75 | 0.776 |
| 6 months vs 3 months treatment | -0.27 | -1.27, 0.72 | 0.589 |

**Abbreviations**: 95% CI, 95% confidence interval; BMI, body mass index.
